# Study on the Interaction of Plasma-Polymerized Hydrogel Coatings with Aqueous Solutions of Different pH

**DOI:** 10.3390/gels9030237

**Published:** 2023-03-17

**Authors:** Monique Levien, Zahra Nasri, Klaus-Dieter Weltmann, Katja Fricke

**Affiliations:** 1 Leibniz Institute for Plasma Science and Technology (INP), 17489 Greifswald, Germany; 2 Center for Innovation Competence Plasmatis, Leibniz Institute for Plasma Science and Technology (INP), 17489 Greifswald, Germany

**Keywords:** hydrogel coatings, atmospheric-pressure plasma polymerization, cyclic voltammetry, modified electrodes

## Abstract

Amphiphilic hydrogels from mixtures of 2-hydroxyethyl methacrylate and 2-(diethylamino)ethyl methacrylate p(HEMA-co-DEAEMA) with specific pH sensitivity and hydrophilic/hydrophobic structures were designed and polymerized via plasma polymerization. The behavior of plasma-polymerized (pp) hydrogels containing different ratios of pH-sensitive DEAEMA segments was investigated concerning possible applications in bioanalytics. In this regard, the morphological changes, permeability, and stability of the hydrogels immersed in solutions of different pHs were studied. The physico-chemical properties of the pp hydrogel coatings were analyzed using X-ray photoelectron spectroscopy, surface free energy measurements, and atomic force microscopy. Wettability measurements showed an increased hydrophilicity of the pp hydrogels when stored in acidic buffers and a slightly hydrophobic behavior after immersion in alkaline solutions, indicating a pH-dependent behavior. Furthermore, the pp (p(HEMA-co-DEAEMA) (ppHD) hydrogels were deposited on gold electrodes and studied electrochemically to investigate the pH sensitivity of the hydrogels. The hydrogel coatings with a higher ratio of DEAEMA segments showed excellent pH responsiveness at the studied pHs (pH 4, 7, and 10), demonstrating the importance of the DEAEMA ratio in the functionality of pp hydrogel films. Due to their stability and pH-responsive properties, pp (p(HEMA-co-DEAEMA) hydrogels are conceivable candidates for functional and immobilization layers for biosensors.

## 1. Introduction

Hydrogels are three-dimensional, cross-linked networks of one or more polymerized monomer structures. Because of their network with hydrophilic groups, they are able to absorb and release water up to several times their own mass [1]. Depending on their composition, hydrogels can respond to external stimuli from the surrounding environment. In particular, smart pH-sensitive hydrogels can reversibly alter their physical and chemical properties according to environmental changes. They are able to respond by increasing or decreasing their absorbency and/or mechanical properties. Depending on the specific chemistry, they can swell or shrink in response to a pH change. Additionally, their water-filled structure gives them a soft, gel-like consistency, and their responsiveness properties make them attractive for biomedical and other applications, such as drug delivery [2,3,4] and tissue engineering [5,6] as well as biosensors and microelectromechanical systems [7,8,9]. Moreover, in combination with electrodes, hydrogels offer the opportunity to be used as bioelectronic/biosensor interfaces, which enhance the communication between biological systems and electronic devices [10,11]. Nevertheless, despite the widespread opportunities of using hydrogels in the field of biosciences, the desired functional and structural properties for interfacing with such biological systems are still challenging. As electrochemical biosensor properties depend on the overall sensing surface, an enlarged electrode area enabling the transfer of ions and electrons is desirable. A large active surface area can be achieved, for instance, by roughening the electrode, by surface patterning, or by generating three-dimensional structures on the electrode that provide architectures with dimensions from a few hundred nanometers to micrometers [12,13,14]. At the same time, an enlarged surface area in combination with functional groups provides excellent binding capabilities for biomolecules [15,16]. Furthermore, an important aspect that has to be considered is the interaction between the hydrogel coating and the surrounding environment. Carrying out studies on morphology, responsiveness, wettability, and stability in aqueous solutions of different pHs is essential for a better understanding of the behavior in biological systems to estimate the applicability in the biomedical field.

Hydrogels synthesized from hydrophilic acrylate monomers gained a lot of attention in the last decades due to their excellent biocompatibility as well as their stimuli-responsive behavior [17]. The most widely used hydrogel backbone is 2-hydroxylethyl methacrylate (HEMA) because of its chemical and hydrolytic stability [17,18]. pH-responsive hydrogels constitute a major branch of responsive hydrogels, which have attracted tremendous research and interest, especially for medical applications. For pharmaceutical applications, for example, hydrogels are of interest as tailor-made carriers for drug release. The pH responsiveness is associated with the functional groups present in the hydrogel polymer backbone, which enable the charge density redistribution of the hydrogel when the pH changes in the surrounding medium. Therefore, by adding an ionic, pH-responsive monomer to the conventional HEMA, the pH-dependent behavior of the hydrogel can be adjusted. Lesho et al., for example, described a swelling behavior with different pH values, due to the ratio of non-ionizable to ionizable groups of N,N-dimethylaminoethyl methacrylate (DMA) [19]. Another well-known pH-responsive monomer is the polybase-containing copolymer 2-(diethylamino)ethyl methacrylate (DEAEMA) with a pK_a_ of 7.3 [20,21,22]. DEAEMA is a smart pH and thermo-responsive polymer with a unique performance upon pH changes. The tertiary amine groups protonate under acidic conditions, whereby fixed charges are formed within the polymer network, resulting in electrostatic repulsive forces and a stretched conformation, enabling pH-dependent swelling or de-swelling [19,23].

Conventionally, hydrogels are prepared via free radical polymerization, photochemical coupling, or 3D printing techniques, including several fabrication steps using chemical detergents for polymerization initiation or cross-linking [24,25,26]. Another approach for the fabrication of hydrogels is the use of atmospheric-pressure plasma polymerization, as described in our previous study [27]. This method is based on surface activation by argon (Ar) plasma, followed by the deposition of an acrylate-based monomer mixture in the liquid phase on the plasma-activated substrate and subsequent polymerization by atmospheric-pressure plasma. It should be underlined that this method eliminates the use of cross linkers, solvents, or other chemicals.

The presented work studies the effects of different pHs on the properties of pp hydrogel coatings and enables a better understanding of how hydrogel coatings can be engineered to serve as reliable tools suitable for sensing in diverse environments. The morphology of the deposited layers was examined on smooth substrates (Si wafer and glass), and, thus, a basic understanding of the deposition of the plasma-polymerized hydrogels was achieved. With regard to a functional layer for sensor applications, investigations on gold electrodes were continued. Hydrogel coatings with different coating thicknesses were generated to investigate the pH-dependent volume phase transition due to ionizable DEAEMA and HEMA as backbone-forming polymers. The scope of this paper is to study the properties of plasma-polymerized hydrogel films of different (p(HEMA-co-DEAEMA)mixture ratios and layer thicknesses in contact with aqueous solutions of different pHs. Additionally, the morphological fold formation and the wettability were analyzed in detail.

## 2. Results and Discussion

### 2.1. Characterization of the Hydrogel Coating

**Figure 1:**
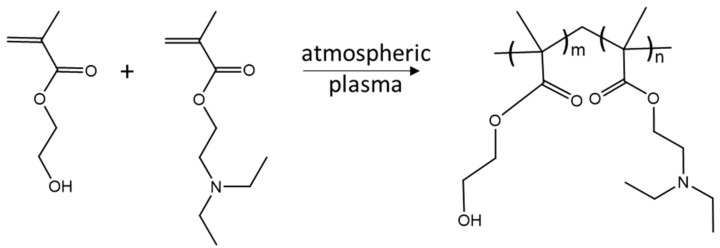
Synthetic route for the plasma polymerization of (p(HEMA-co-DEAEMA) films.

Atmospheric-pressure plasma provides high concentrations of chemically active species such as free electrons, reactive oxygen and nitrogen species (e.g., •OH, ^1^O_2_, H_2_O_2_, ^•^O_2_^−^, ^•^O, O_3_), ions, light radiation, and other neutral molecules. The pre-treatment of the substrate with plasma leads to a plasma-activated substrate [28]. Through this, the wettability of the surface is improved, and the monomer mixture spreads on the activated substrate, which a subsequent plasma polymerization step can follow. The resulting plasma polymer coatings (Figure 1) are generally highly cross-linked, with an excellent level of adhesion to any surface and a high level of stability [29].

First, the pp HD coatings were deposited on silicon wafers to show the deposition behavior. The generation of thin coatings through the nebulizer method revealed thicknesses of several hundred nanometers, while the coatings produced by using the droplet method exhibited layer thicknesses almost twice the layer thickness (Table 1). Due to the gelation properties of HEMA, it has been widely used as the backbone for hydrogels [18,30]. As a result of plasma polymerization with different mixture ratios, the coating thickness increases, and more of the “backbone forming” unit is added to the pp (p(HEMA-co-DEAEMA) mixture [31].

**Figure 2:**
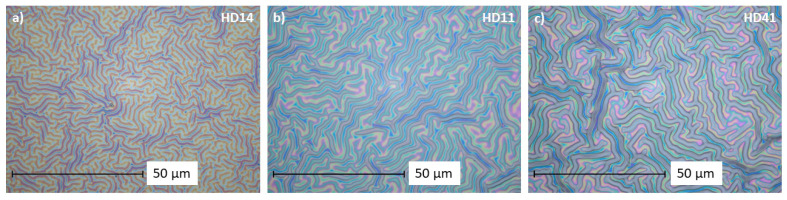
Microscopic images of the hydrogel films (**a**): d-HD14, (**b**): d-HD11, and (**c**): d-HD41 at 1500× magnification.

Microstructuration based on surface instabilities fabricated by an atmospheric-pressure plasma jet has been reported recently [27]. Figure 2 shows the microscopic images of the hydrogel mixtures generated with the droplet method. The effect of plasma on the liquid monomer mixture and the resulting polymer causes characteristic wrinkle structures, which were studied before and after storage in water using layer thickness and wrinkle width measurements (Table 1 and Figures S1 and S2).

As can be seen from the pictures (Figure 2), the wrinkle width increases the higher the HEMA portion of the mixture. The same trend was observed for n-HD hydrogels (Figure S3). The corresponding measurements (Figures S1 and S2) confirm the observations; the wrinkles from d-HD14 are the smallest at 1.3 ± 0.18 µm, and the wrinkles of the d-HD41 mixture are the widest at 2.2 ± 0.19 µm. It is apparent that an increment of HEMA in hydrogel coatings can cause more pronounced wrinkles. Often, wrinkling is a result of two materials having different physicochemical properties. While the surface tension of DEAEMA is 28.9 dyne/cm, the value for HEMA is slightly higher at 33.7 dyne/cm [32]. Films become stiffer with increased concentrations of HEMA, leading to more pronounced wrinkles when a certain stimulus is applied for creating surface instabilities [33]. Both generation methods show a good stability in terms of wrinkle width and thickness of the hydrogels after storage in water (see Figures S2 and S3), only for n-HD 11 and n-HD41, a noticeable decrease in the thickness was observed.

**Figure 3:**
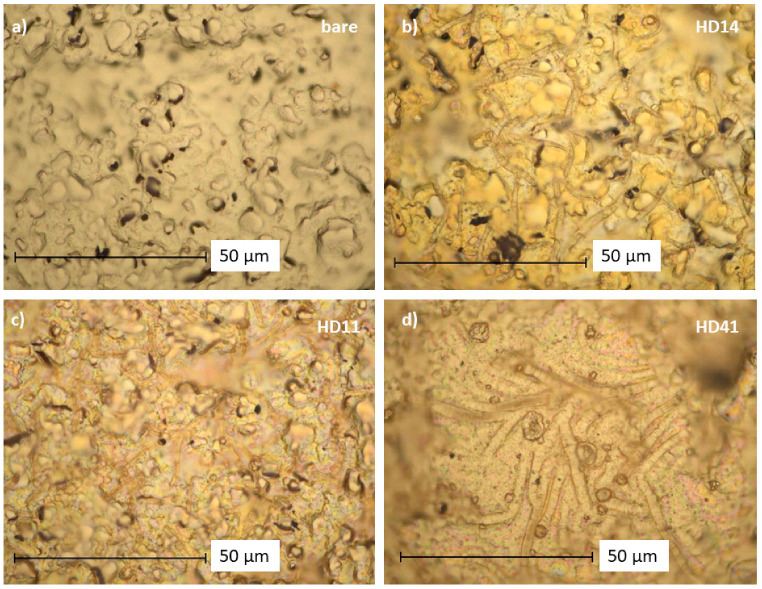
Optical microscope images (1500× magnification) of (**a**): the unmodiefied goldelectrode and the plasma-polymerized hydrogel coatings (**b**): HD14, (**c**): HD11, (**d**): HD41) deposited on gold, generated through the droplet method.

A transfer of the pp HD mixture ratios on gold electrodes is displayed in Figure 3. In comparison to the unmodified bare gold electrode (Figure 3a), the pp hydrogel coatings cover the valleys and bumps of the roughly structured electrode, and reveal a filament structure formation with colorful areas (Figure S4) of the deposits. For pp HD14 (Figure 3b), the filament formation is more pronounced, whereas pp HD11 and pp HD41 (Figure 3c,d) show blurred areas, indicating a more dense hydrogel network on the electrode.

**Figure 4:**
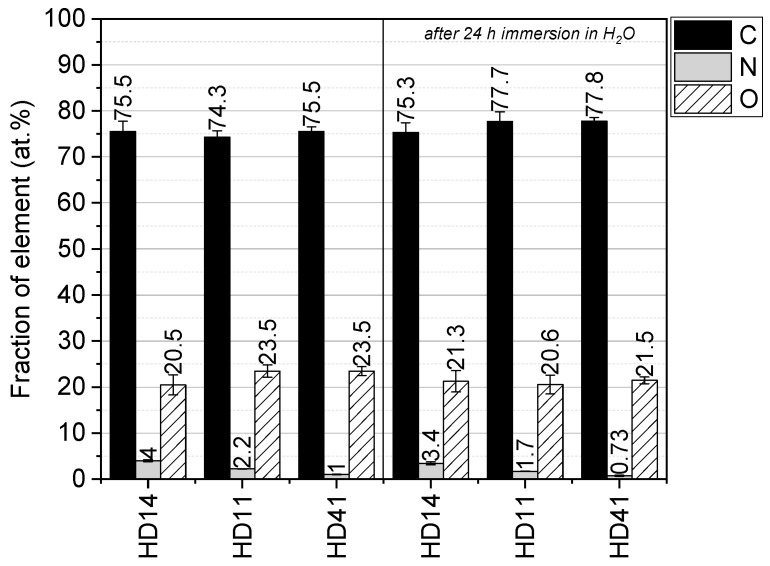
XPS elemental analysis of the different hydrogel mixtures (d-HD), as-deposited and after immersion in water for 24 h (*n* = 3).

The interaction of reactive plasma species with the (p(HEMA-co-DEAEMA) mixture induces a variety of chemical and physical processes, whereby functional groups can be retained or introduced. XPS measurements were carried out to analyze the elemental composition before (as-deposited) and after 24 h immersion in deionized water (Figure 4). The plasma-polymerized hydrogels were composed of carbon (74–77 at. %), oxygen (20–23 at. %), and nitrogen (≤4 at. %). Figure 3 reveals that the fraction of oxygen and nitrogen depends on the pp (p(HEMA-co-DEAEMA) mixture ratio. An increase in the nitrogen content with a rising DEAEMA portion was observed. For coatings immersed in deionized water for 24 h, a partial loss of oxygen and nitrogen can be seen. This loss might be attributed to the removal of residual oligomers that are not polymerized. Nevertheless, the results demonstrate good chemical stability of the hydrogel films in aqueous media.

The surface wettability was determined by conducting water contact angle (WCA) measurements via the sessile drop method on the pp hydrogels dried after immersion in neutral (pH 7), acidic (pH 4), and alkaline (pH 10) buffer solutions (Table 2). The bare gold electrode is characterized by a hydrophobic surface with a water contact angle of ~94°; the pp hydrogels on the SPE dried from different pH solutions demonstrate significant contact angle variations in the range of 27° to 77°. For d-HD14, the measured contact angles were 36° and 77° after drying from solutions of pH 4 and pH 10, respectively. This hydrophilic to more hydrophobic trend after drying from solutions of higher pH was observed for all mixture ratios of the d-HD coatings. The increase in the wettability of the hydrogel films is caused by the protonation of the tertiary amine moieties of DEAEMA in the mixtures at pH 4 and pH 7, since for DEAEMA, the pK_a_ is ≈7.3 [22,34,35]. This allows the absorption of larger amounts of water, leading to smaller water contact angles [36,37]. For the thin coatings, n-HD, the trend is comparable, but compared to their thicker counterparts, n-HD films were found to be more hydrophilic. Overall, the water contact angle measurements indicate a different behavior of the pp HD coatings depending on the pH.

**Figure 5:**
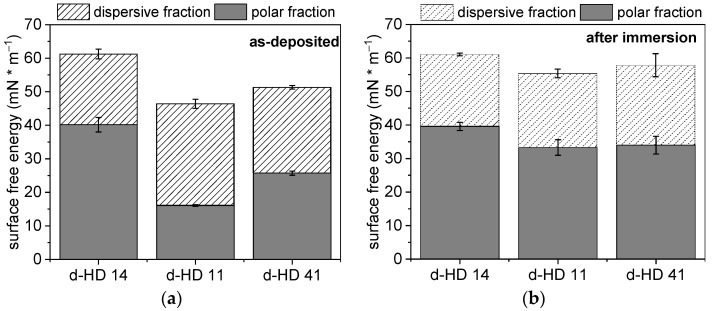
Surface free energy of the hydrogel mixtures generated using the droplet method (**a**) as-deposited and (**b**) after immersion in water for 24 h, divided into dispersive and polar fraction.

The surface free energy (SFE) is the work that is needed to increase the surface area of a solid phase. Since every system strives for a state of low free energy, solids form an interface with liquids in order to reduce this energy. Moreover, the SFE plays a key role in cell material interactions in terms of tissue engineering or biosensor development [38,39]. For the calculation of the SFE, the method of Owens, Wendt, Rabel, and Kaelble [40] was used, whereby the SFE is divided into a polar and a dispersive component Figure 5 shows the SFE for the different d-HD films as-deposited and after immersion in deionized water for 24 h. The bare gold electrode has an SFE of 30 mN/m^−1^, consisting of 3.6 ± 1.3 mN/m^−1^ polar fraction and a dispersive fraction of 27.2 ± 1.6 mN/m^−1^ (Figure S5). With deposited hydrogel coatings on the electrode surfaces, the SFE increases, ranging from 45 to 60 mN/m^−1^, due to a remarkable increase in the polar fractions of the hydrogel mixtures (Figure 5a). After storage in water (Figure 5b), a further increase in the SFE, especially for the polar fraction, was recorded for d-HD11 and d-HD41, whereby d-HD14 remains at the same level. This can be explained by the introduction of polar groups (-OH) to the pp hydrogel films during the immersion, resulting in an increased polar fraction [41]. The polar fraction for d-HD14 is comparably high before and after immersion. For all HD mixtures studied, the SFE ranges between 55 and 61 mN/m^−1^ after immersion, which is in agreement with the literature values for HEMA-based hydrogels [42]. In contrast, the SFE of n-HD films are comparably lower (~45 mN/m^−1^) and do not change significantly after immersion in water (Figure S6). Moreover, a lower polar fraction was determined for the n-HD coatings after immersion with 15 mN/m^−1^ in comparison to the d-HD coatings with 35 mN/m^−1^. Since both coating generation methods (droplet and nebulizer) have the same polymerization time of 30 s, the thinner n-HD coatings might polymerize more strongly in the same time, which leads to lower proportions of the functional polar groups. A similar phenomenon was observed by Klages et al., where a higher degree of functional groups from the polymerization of glucidyl methacrylate remains due to shorter plasma treatment times [43]. The results confirm high SFE and sorption properties due to a high value of polar forces on the plasma-polymerized hydrogels generated via the droplet method.

To study the presence of pH-responsive behavior of the pp hydrogels, free surface energy measurements were performed for HD coatings and bare gold electrodes, stored for 24 h in buffer solutions of pH 4, 7, and 10, respectively [44].

**Figure 6:**
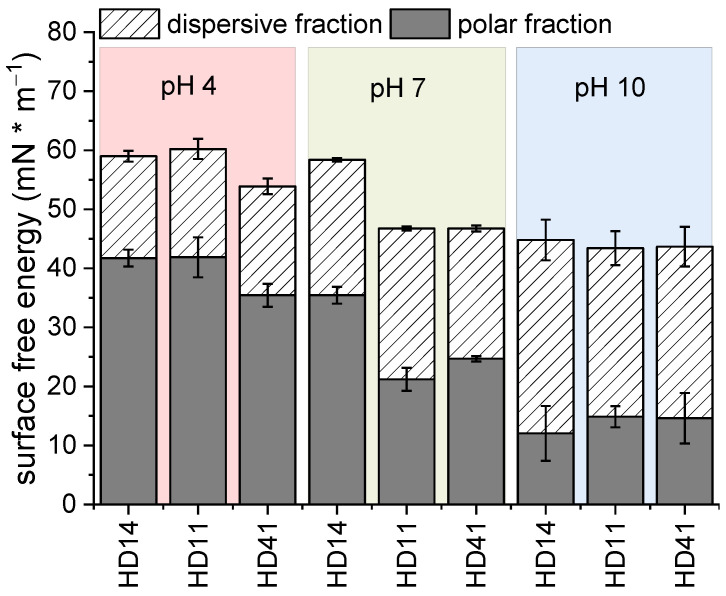
Surface free energy of the pp d-HD films stored in different pH solutions.

**Figure 7:**
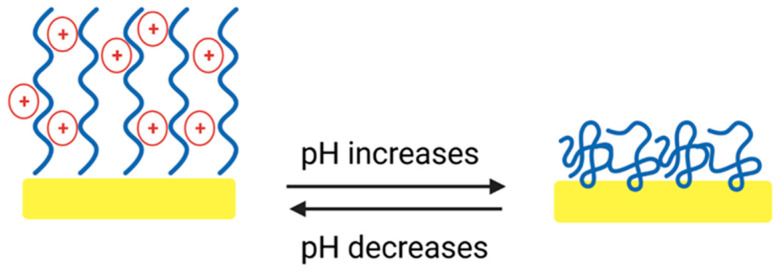
Schematic representation of the plasma-polymerized (p(HEMA-co-DEAEMA) films at different pHs. Created with BioRender.com.

For the bare gold electrodes, the overall SFE increases after storage in pH 4 and 10 to 50 and 57 mN/m^−1^, respectively (Figure S5). Additionally, a high polar fraction was noticed, 33 mN/m^−1^ at pH 4 and 46 mN/m^−1^ at pH 10. This can be attributed to the presence of acid and alkaline molecules that remain on the gold surface after storage in the different pH buffer solutions. For all d-HD coatings investigated, it can be seen that the polar component decreases with increasing pH (Figure 6). At pH 4, the tertiary amine groups of DEAEMA are protonated, leading to an electrostatic repulsion of the positive charges, which results in a stretched, flexible conformation of the HD14 coating (Figure 7) [45,46,47].

The polar groups of the inner network become more accessible, which allows a stronger absorption of polar solutions (such as water and ethylene glycol). Hence, the contact angles of the liquids used are smaller, leading to higher polar fractions [48]. Since for DEAEMA the pK_a_ is ≈7.3, this phenomenon is partly valid for pH 7. For d-HD14, a slight decrease in the polar component was observed, whereas for d-HD11 and d-HD41, a remarkably lower SFE was observed along with a smaller portion of the polar fraction, demonstrating an “intermediate state” behavior of these mixtures, since the degree of ionized tertiary amines seems to be lower [48]. At pH 10, DEAEMA becomes deprotonated, so the coatings become more hydrophobic, resulting in lower polar fractions. In terms of the hydrogel films generated with the nebulizer method (Figure S7), only in the case of the HD41 mixture was the trend towards lower SFE with increasing pH measured. Stratakis et al. [49] observed a comparable polyampholyte behavior, showing a reversible switch of superhydrophilicity at low pH values and superhydrophobicity at higher pH values of acrylate-based surfaces. In conclusion, the plasma-polymerized hydrogels demonstrate pH-responsive behavior, depending on the mixture ratio and coating thickness (Figure 7). On the basis of these results, the following investigations on pp hydrogel films were focused on d-HD films, due to the pronounced changes in the behavior at different pHs.

### 2.2. Surface Topography

**Figure 8:**
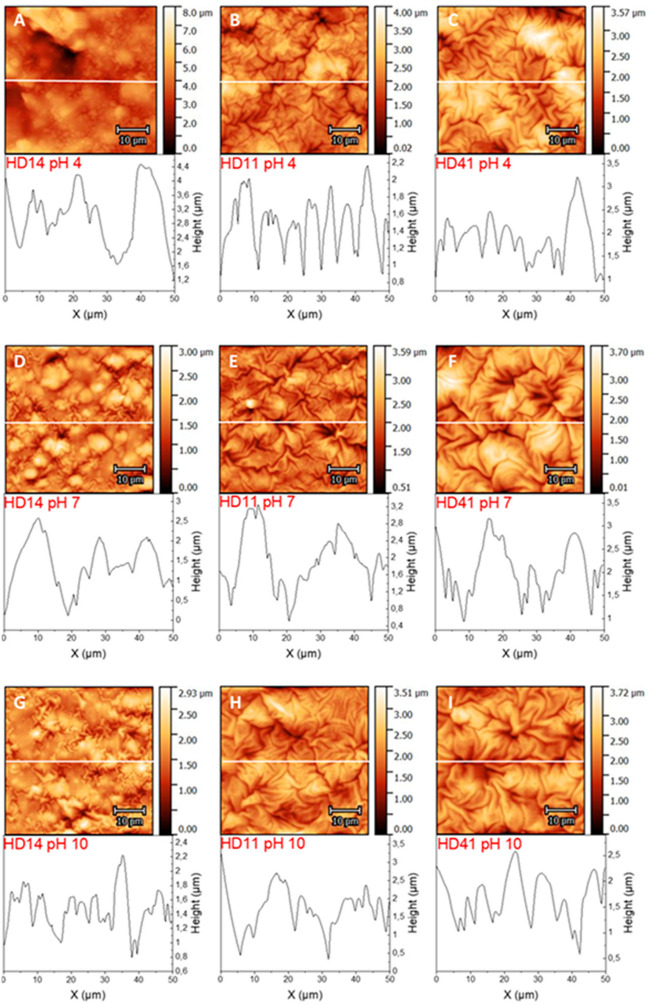
(**A**–**I**): AFM images and the corresponding height profiles of the plasma-polymerized hydrogel coatings (d-HD14, d-HD11, and d-HD41) in acidic (pH 4) (**A**–**C**), neutral (pH7) (**D**–**F**), and alkaline (pH 10) (**G**–**I**) solutions. White lines indicate the location of the height profile. AFM images of the coatings in air and a bare gold electrode are shown in Figures S9 and S10.

AFM was applied to study the topography of the plasma-polymerized d-HD films. Deposition on smooth glass substrates (Figure S8) revealed network-like structures for HD14, while with increasing HEMA content, a fold structure is pronounced. For HD41, it was observed that some areas of the folds seem to stick together. A transfer of the hydrogel films onto gold electrodes was examined in air (Figure S9) and immersed in solutions of different pHs (Figure 8A–I). For the HD films exposed to air only (Figure S9), it can be seen that surface instabilities during the plasma polymerization processes also induce the formation of folds, due to mechanical stress or monomer evaporation during the plasma process [50,51]. For d-HD14, the folds are not strongly pronounced, and at the same time, the surface shows smooth areas, and the folds of d-HD11 and d-HD41 appear more tightly packed [33,37]. Furthermore, the RMS surface roughness was determined (Table 3), which reveals that each hydrogel is highly rough, but in comparison to the bare gold electrode (RMS of 0.89 ± 0.02 µm, AFM see Figure S10), the coatings have a smoothening effect on the surface and irregularities are covered (as was shown in the microscopic pictures before). Nevertheless, the formed folds of the hydrogel coatings are irregular as well, as can be seen in the corresponding height profiles below each AFM image. HEMA is well known as the “backbone-forming” unit of acrylate-based hydrogels; therefore, the addition of more HEMA in the mixture (while keeping the process parameters constant) leads to a stronger fold formation, which correlates with the surface thickness [30,31,52]. For d-HD films immersed in buffer solutions of different pHs, morphological changes can be seen for d-HD14 as compared d-HD11 and d-HD41. In pH 4 and pH 7, the surface features of d-HD14 with small fold structures disappear, and the height of the surface features increases enormously, indicating a swollen state (Figure 7). Since the tertiary amine groups of DEAEMA are protonated in aqueous media, a stretched conformation (as mentioned in the SFE section) of the d-HD14 coating occurs [34,35,45], due to the absorption of more water molecules (as previously shown in the wettability and SFE experiments). In particular, in the buffer solution of pH 4, the RMS increased by a factor of 1.7, demonstrating significant differences in the heights of the topographical features. The AFM image of d-HD14 stored at pH 10 shows smaller folded structures than at pH 4 and pH 7. Furthermore, the RMS of 0.83 ± 0.04 µm is higher than in pH 7 and lower than in pH 4, which indicates a shrunken state due to the deprotonation of the tertiary amine groups. For d-HD11 and d-HD41 films, the amount of DEAEMA is lower, resulting in a limited ionization level and less intense swelling behavior at an acidic pH. While the RMS roughness of d-HD11 and d-HD41 stored at pH 4 and pH 7 are similar, the value increases after immersion in pH 10, demonstrating again the shrunken surfaces with structural changes resulting in strongly pronounced folds. The results of the AFM measurements clearly show that the H:D mixture ratio has a direct influence on the morphology of the surface texture. To test the influence of the pH value on the surface morphology, AFM was performed at acidic as well as at alkaline conditions, revealing a remarkable response attributed to the DEAEMA amount. Similar observations of spin-coated HEMA and poly(ethylene glycol) diacrylate (PEGDA) hydrogels were made by González-Henríquez et al. [37]

### 2.3. Electrochemical Characterization

**Figure 9:**
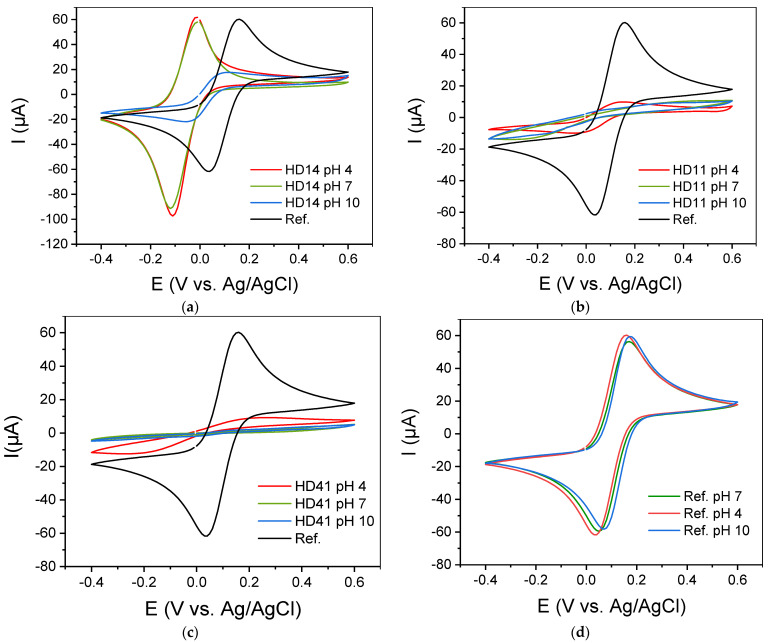
Cyclic voltammograms (**a**–**c**) of the plasma-polymerized d-HD films acquired in buffer solutions of different pHs, (**d**): [Fe(CN)_6_]^3−/4−^ at the bare gold electrode in solutions of different pHs. CV recordings of n-HD films in different pHs are shown in Figure S11.

In general, hydrogels’ permeability to ions makes them attractive as matrices for electrochemical mediators. Therefore, to evaluate the applicability of the pp d-HD films as electrode interfaces, cyclic voltammograms of [Fe(CN)_6_]^3−/4−^ as the redox probe in the presence of pp d-HD films were recorded. Figure 9a–d presents cyclic voltammograms of 5 mM [Fe(CN)_6_]^3−/4−^ for the unmodified bare gold electrode compared to those obtained for pp d-HD-coated electrodes in buffer solutions of different pHs. In comparison to the bare gold electrode, which served as the basic material, a clearly enhanced conversion of the redox ions is found for the gold electrode coated with pp d-HD14 at pH 4 and pH 7 (Figure 9a). While the bare gold electrode has a peak current of 60 µA and −60 µA (anodic and cathodic, respectively), ppHD 14 at pH 4 and pH 7 shows a significant increase in the reductive peak current to −100 µA. This is attributed to the pH-responsive property of the DEAEMA parts in the hydrogel, as the CVs of [Fe(CN)_6_]^3−/4−^ from the bare gold electrode showed no pH-dependent behavior (Figure 9d) [53]. In acidic conditions, the polymer chains within the d-HD14 hydrogel stretch due to the electrostatic repulsion between the positive electrical charges [34,45] (Figure 7). The results are in correlation with the AFM images of d-HD 14 films stored at pH 4 (see previous section), where surface characteristic features exhibited an enormous increase in height and roughness. As the redox probe [Fe(CN)6]^3−/4−^ has negative charges, it will accumulate on the enlarged surface of the protonated and positively charged DEAEMA polymer chains. The reduced species [Fe(CN)6]^4−^ can bind more strongly to the polymer backbone due to bridging protons, resulting in a higher reductive current response. This phenomenon was also reported by Wang et al. [54].

Additionally, the potential of the pp/SPEs shifts from 0.03 V vs. Ag/AgCl reference electrode to −0.12 V. This shift in the potential reflects the rate of electron transfer and indicates increasing difficulty for the electron transfer reaction between the redox probe and the electrode with increasing thickness of the film. Moreover, pp films can be considered as membranes exhibiting permselectivity properties. In the case of pp (p(HEMA-co-DEAEMA) films, they act as an anion exchange membrane permitting the transport of [Fe(CN)6]^4−^ ions into the highly positively charged brush environment and hindering the transport of K^+^ species (Donnan exclusion). This exclusion process due to the permselectivity properties introduces the presence of a Donnan potential, which affects the formal potential of the redox centers [45]. This interfacial electrical potential is, therefore, related to the presence of pH- dependent fixed charges in the coating, as described by Anson [55,56] and Doblhofer [57,58]. For pH 7, the same phenomenon was observed, but with less intensity (as also discussed in the section on surface free energy and pH-responsive behavior). However, for pH 10, the peak current decreases due to the deprotonation of the DEAEMA, resulting in a more hydrophobic property (as can be seen from the free surface energy results above). The electrostatic repulsion between the positive charges on the polymer chains is weakened or has disappeared completely, and the attraction between the polymer chains increases in such a way that the polymer chain coils and, thus, collapses [23]. The collapsed and compact structure of pp d-HD films at pH 10 leads to a lack of flexibility of the coating, which results in the suppression of the electroactive species transmission through the hydrogel to the electrode surface [34]. The same trend was observed for d-HD 11, as can be seen in Figure 9b, but with a strikingly low response. While the current peaks can still be seen at pH 4, they are almost flattened at pH 7 and completely lost at pH 10. This behavior can be explained by the lower DEAEMA content and, therefore, a lower protonation level, leading to a less expanded conformation at lower pHs. The influence of a very low DEAEMA content (HD41) becomes even more obvious in Figure 9c, where no oxidation and reduction peaks can be seen at different pH values. This confirms that by increasing the DEAEMA content, the electrostatic adsorption of the negatively charged redox probe on the hydrogel is enhanced, which produces a higher current in the related CV. In Figure S11, the CVs for the n-HD hydrogels are shown. The trend in different pH solutions for the films is the same as for the d-HD hydrogels, but with strikingly less response. This might be attributed to a higher degree of polymerization (as discussed in the SFE section) of the thinner coatings, resulting in a lesser electron transmission and entrapment capacity.

## 3. Conclusions

In this study, pH-sensitive plasma-polymerized hydrogel coatings composed of different (p(HEMA-co-DEAEMA) mixtures were deposited on gold electrodes and investigated. The copolymerization of HEMA and DEAEMA with varying volume ratios exhibits various surface properties. The results indicated that the pp (p(HEMA-co-DEAEMA) with a higher portion of DEAEMA showed pH-responsive behavior. The responsive properties of this hydrogel are due to the outstanding protonation capacity of the pendant tertiary amine groups of the DEAEMA segment. At neutral and acidic conditions, pDEAEMA is water soluble and hydrophilic, generating swollen capabilities in the hydrogel network. In contrast, this polymer becomes relatively insoluble, with slightly hydrophobic behavior, at alkaline pHs. In this regard, the protonation capacity of the tertiary amines of pDEAEMA has an influence on the morphology, the wettability, and the electron transport properties through the hydrogel coatings. The surface of the HD films is characterized by a folded morphology, which is generated during the plasma polymerization. Our studies showed that hydrogel coatings generated with the droplet method possess excellent stability after storage in water. A change in the surface wettability, after the hydrogels were dried from different pH solutions, was found for every mixture, whereby a trend from hydrophilic to hydrophobic after drying from pH 4 and pH 10 was observed, respectively. Furthermore, the polar fraction as part of the free surface energy decreases enormously for all coatings when dried from a solution of pH 10. For hydrogel films generated with the nebulizer method, this trend was absent. Plasma-polymerized hydrogels with a thickness of ~0.5–1 µm and above might polymerize not as strongly as the thinner hydrogel coatings from the nebulizer method, resulting in a higher polar component and a more pronounced responsive behavior towards different pH solutions. This leads to the assumption that such coatings are very well suited for biological/medical applications and introduces d-HD14 hydrogel as a promising environment for immobilizing biomolecules.

## 4. Materials and Methods

### 4.1. Chemicals and Materials

HEMA (2-hydroxyethyl methacrylate) and DEAEMA (2-(diethyl amino) ethyl methacrylate), both 98%, were purchased from Sigma Aldrich, Germany, and used as received. K_4_[Fe(CN)_6_]*3H_2_O Potassium hexacyanoferrate (II) trihydrate and K_3_[Fe(CN)_6_] Potassium hexacyanoferrate (III), both with 99% purity, were purchased from Carl Roth GmbH, Germany. NIST (National Institute of Standards and Technology) traceable buffer solutions of pHs 4.01, 7.01, and 10.01, were purchased from Hanna Instruments GmbH, Germany. Gold screen-printed electrodes (DropSense 220 AT, Metrohm, Filderstadt, Germany) were used as substrates.

### 4.2. Plasma Source and Plasma Polymerization

**Figure 10:**
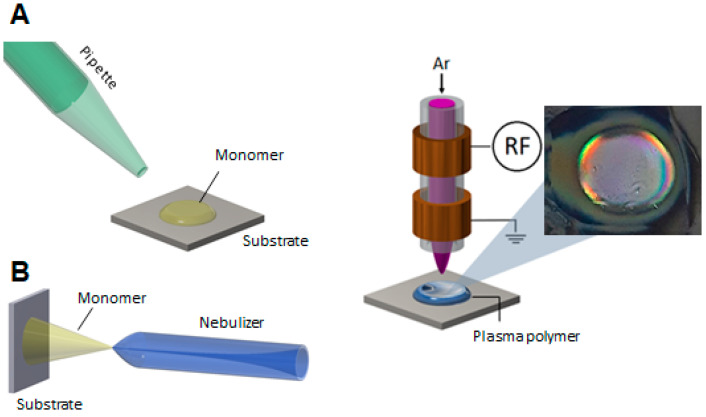
Schemes of two setups used for the synthesis of hydrogel films of different thickness ((**A**): droplet method, (**B**): nebulizer method) and of the atmospheric-pressure plasma jet applied for the polymerization of (p(HEMA-co-DEAEMA) films, and a photograph of a pp hydrogel film.

The deposition of the hydrogel coatings comprises three consecutive steps: (i) pre-treatment of the gold surface by using Ar plasma for 30 s; (ii) applying the liquid phase monomer mixture (HEMA-co-DEAEMA, HD, with different mixture ratios 1:4, 1:1, and 4:1 *v*/*v*) on the gold substrate, and (iii) subsequent plasma polymerization by applying Ar plasma for 30 s. Plasma-polymerized hydrogel coatings of different thicknesses were generated by using two setups schematically shown in Figure 10. The droplet method was used for the deposition of coatings with thicknesses >0.5 µm by pipetting 0.1 µL of the HD mixture onto the plasma-activated gold substrate, whereas the nebulizer method (1 standard liter per minute (slm) Ar flow rate and 1 s nebulization time) was applied for depositing a thin liquid layer of the HD mixture, resulting in coatings with thicknesses <0.5 µm. The plasma-induced surface activation and polymerization of the HD mixtures were carried out by using a 27.12 MHz-driven atmospheric-pressure plasma jet (described in detail by Schaefer et al. [59]), schematically shown in Figure 1, operated at a plasma input power of 5 W, with 1 slm Ar as working gas at a nozzle outlet-to-substrate distance of 5 mm.

According to the methodology applied, the obtained films are referred to as d-HD or n-HD when the droplet (d) or nebulizer (n) setup was used.

### 4.3. Surface Characterization

The elemental composition of the coatings was characterized through high-resolution scanning X-ray photoelectron spectroscopy (XPS) using an Axis Supra DLD electron spectrometer (Kratos Analytical, Manchester, UK) with a monochromatic Al Kα source (1486.6 eV). The instrument was set to the medium magnification (field of view 2) lens mode and the slot mode, providing an analysis area of approximately 250 µm in diameter. Data acquisition and processing were carried out using CasaXPS software, version 2.14dev29 (Casa Software Ltd., Teignmouth, UK). Concentrations are given in atomic percent (at. %).

Atomic force microscopy (AFM) (coreAFM, Nanosurf, Switzerland) was used to examine the surface morphology of the obtained hydrogel coatings, as well as the root mean square surface roughness. AFM images were acquired in non-contact mode by using a PPP-XYNCHR tip with a spring constant of 42 N/m. Measurements in solutions of different pHs (4.01, 7.01, and 10.01) were performed with a 150Al-G tip (spring constant 5 N/m). AFM images were processed using the Gwyddion freeware (Version 2.55; 2019) [60].

To determine the surface free energy (SFE) of the coatings, the contact angles of three different liquids, namely millipore water, ethyleneglycol, and diiodomethane, were measured using the OCA 30 system and SCA20 software from DataPhysics Instruments GmbH, Filderstadt, Germany. A sessile drop (0.5 µL) of each liquid was formed using an automatic micro syringe system and applied to the plasma-polymerized hydrogels. After the drop contour was drawn, the surface free energy was calculated by using the Owen/Wendt–Rabel–Erbil (OWR) method [40]. The measurements were performed on films as-deposited, and after 24 h immersion in millipore water or in buffer solutions (Hanna Instruments GmbH, Vöhringen, Germany), respectively. After immersion, the coatings were dried for 6 h in air.

A surface profiler (Alpha-Step D-600 Surface profiler from KLA-Tencor, Milpitas, CA, USA) was used to measure the thicknesses of the hydrogel films. The stylus records the profile of the plasma-polymerized hydrogel over a length of 10 mm with a force of 5 mg and a speed of 0.10 mm/s. Each measurement was performed in triplicate, and the mean value was calculated (Alpha-Step D-600 Stylus Profiler, Version 4.1.2.0).

Optical microscope measurements were carried out using a 3D Laser scanning microscope VK-X 3000 series (Keyence GmbH, Neu-Isenburg, Germany), and images were processed with Multifile Analyzer (Version 3.3.1.85).

### 4.4. Electrochemical Characterization

The electrochemical properties of the plasma-polymerized pp HD films were investigated through cyclic voltammetry (CV) using a PalmSens4 potentiostat (PalmSens, Houten, The Netherlands). Voltammograms were acquired in a three-electrode cell (refs. 250AT) using a modified screen-printed gold electrode as the working electrode, an isolated Ag/AgCl reference electrode, and a platinum counter electrode. Samples were subjected to a cyclical potential from −300 to 600 mV at a scan rate of 10 mV s^−1^. CV studies of the pp HD films were performed in buffer solutions of pH 4, 7, and 10, in the presence of a 5 mM potassium ferricyanide solution K_3_[Fe(CN/K_4_[Fe(CN)_6_] (1:1) as the redox probe.

## Figures and Tables

**Table 1:** Thickness of the hydrogel coatings generated by the nebulizer (n-HD) and droplet method (d-HD), respectively. (*n* = 3).

	n-HD Nebulizer Method (µm)	d-HD Droplet Method (µm)
**HD14**	0.1 ± 0.00	0.5 ± 0.03
**HD11**	0.4 ± 0.06	1.1 ± 0.16
**HD41**	0.6 ± 0.02	1.5 ± 0.14

**Table 2:** Water contact angles of the plasma-polymerized hydrogels after immersion in buffer solutions of pH 4, 7, and 10 for the different pp HD mixture ratios. (*n* = 3).

	d-HD14	d-HD11	d-HD41	n-HD14	n-HD11	n-HD41
**pH 4**	36° ± 5°	42° ± 5°	43° ± 5°	27° ± 6°	28° ± 3°	39° ± 5°
**pH 7**	43° ± 5°	53° ± 1°	56° ± 2°	28° ± 6°	34° ± 2°	54° ± 2°
**pH 10**	77° ± 5°	68° ± 4°	63° ± 3°	42° ± 8°	41° ± 6°	53° ± 4°

**Table 3:** Root mean square surface roughness (RMS) of hydrogels in air and in different buffer solutions. (*n* = 3).

	Air	pH 4	pH 7	pH 10
pp d-HD14 (µm)	0.67 ± 0.05	1.08 ± 0.21	0.65 ± 0.02	0.83 ± 0.04
pp d-HD11 (µm)	0.69 ± 0.05	0.73 ± 0.05	0.78 ± 0.05	1.25 ± 0.20
pp d-HD41 (µm)	0.68 ± 0.02	0.74 ± 0.02	0.79 ± 0.09	1.36 ± 0.24
Bare gold EL (µm)	0.89 ± 0.02			

## Data Availability

Raw data were generated at the Leibniz Institute for Plasma Science and Technology (INP). Derived data supporting the findings are available from the corresponding author [M.L] on request.

## Supplementary Materials

The following supporting information can be downloaded at: https://www.mdpi.com/article/10.3390/gels9030237/s1, Figure S1: Coating thicknesses (*n* = 3) and wrinkle widths (*n* = 10) of the hydrogel mixtures generated through the droplet method, measured before and after storage in water. Figure S2: Coating thicknesses (*n* = 3) and wrinkle widths (*n* = 10) of the hydrogel mixtures (*n* = 10) generated through the nebulizer method, measured before and after storage in water. Figure S3: Microscopic images of the hydrogel films n-HD14, n-HD11, and n-HD41 at 1500× magnification. Figure S4: Optical microscope image (1500× magnification) of the hydrogel coatings HD41 deposited on gold, generated through the droplet method. Figure S5: Surface free energy of the bare gold electrode and the bare gold electrode stored in different pH solutions. Figure S6: Surface free energy of the hydrogel mixtures generated through the nebulizer method (left: as-deposited, right: after immersion in water for 24 h) divided into dispersive and polar fractions. Figure S7: Surface free energy of the n-HD mixtures dried from different pH solutions. Figure S8: AFM images of the plasma-polymerized hydrogel coatings d-HD14, d-HD11, and d-HD41 deposited on a smooth glass substrate. Figure S9: AFM images and the corresponding height profiles of the plasma-polymerized hydrogel coatings d-HD14 (A), d-HD11 (B), and d-HD41 (C) deposited on gold electrodes. Figure S10: AFM image and the corresponding height profile of the bare gold electrode. Figure S11: Cyclic voltammograms (A–C) of the plasma-polymerized hydrogel mixtures (generated through the nebulizer method) in different pH buffer solutions.
