# Supplementary material for: Study on the Interaction of Plasma-Polymerized Hydrogel Coatings with Aqueous Solutions of Different pH
Source: Gels. 2023 Mar 17;9(3):237. doi: 10.3390/gels9030237 (PMC10048005; doi:10.3390/gels9030237)
Supplement: Supplementary file 1 [file gels-09-00237-s001.zip › gels-2259185-supplementary.pdf]

Supporting information

Study on the Interaction of Plasma-Polymerized Hydrogel Coatings with Aqueous Solutions of Different pH

Monique Levien,[1], Zahra Nasri[2], Klaus-Dieter Weltmann[1], Katja Fricke*[1]

[1]Junior Research Group Biosensing Surfaces, Leibniz Institute for Plasma Science and Technology (INP), 17489 Greifswald, Germany
Corresponding author: monique.levien@inp-greifswald.de

[2]Center for Innovation Competence Plasmatis, Leibniz Institute for Plasma Science and Technology (INP), 17489 Greifswald, Germany

Figure S1. Coating thicknesses (n=3) and wrinkle widths (n=10) of the hydrogel mixtures generated by the droplet method, measured before and after storage in water.

	**Coating thickness (µm):**		**Wrinkle width (µm):**	
	as deposited	after storage in H_2O	as deposited	after storage in H_2O
**d-HD14**	0.47 ± 0.03	0.33 ± 0.09	1.3 ± 0.18	1.0 ± 0.15
**d-HD11**	1.05 ± 0.16	1.02 ± 0.11	2.0 ± 0.18	1.5 ± 0.36
**d-HD41**	1.54 ± 0.14	1.42 ± 0.12	2.2 ± 0.19	2.2 ± 0.24

Figure S2. Coating thicknesses (n=3) and wrinkle widths (n=10) of the hydrogel mixtures (n=10) generated by the nebulizer method, measured before and after storage in water.

	**Coating thickness (µm):**		**Wrinkle width (µm):**	
	as deposited	after storage in H_2O	as deposited	after storage in H_2O
**n-HD14**	0.14 ± 0.00	0.15 ± 0.02	No wrinkles	No wrinkles
**n-HD11**	0.40 ± 0.06	0.20 ± 0.01	1.6 ± 0.43	1.4 ± 0.28
**n-HD41**	0.59 ± 0.07	0.40 ± 0.04	1.7 ± 0.14	1.5 ± 0.28

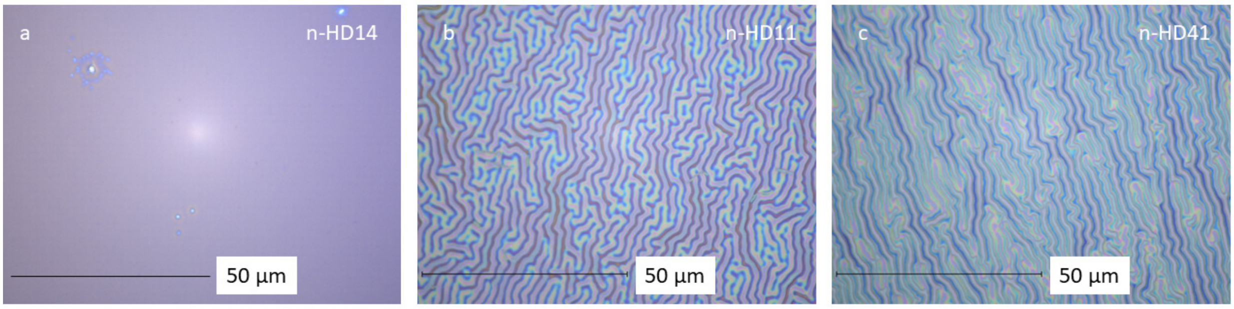

Figure S3. Microscopic images of the hydrogel films n-HD14, n-HD11 and n-HD41 at 1500x magnification.

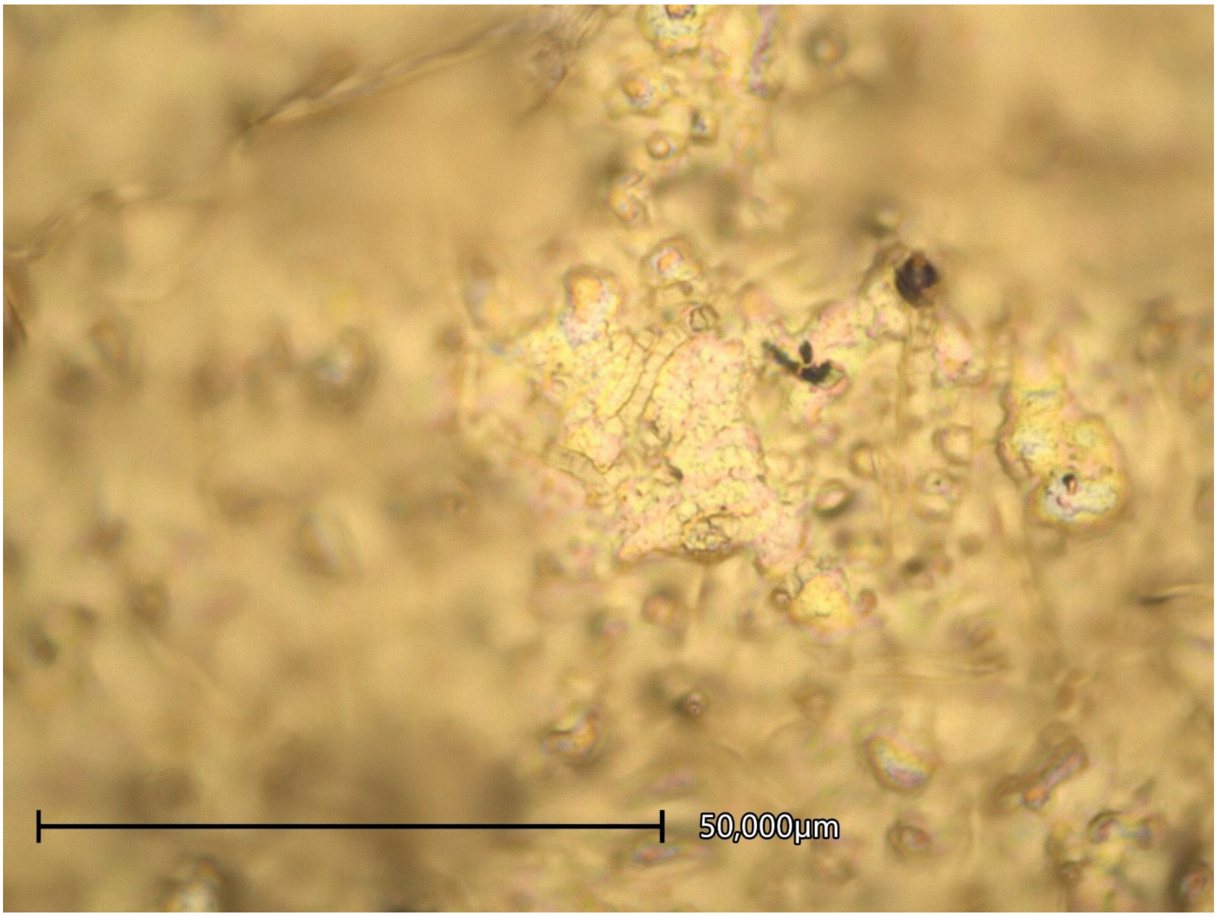

Figure S4. Optical microscope image (1500x magnification) of the hydrogel coatings HD41 deposited on gold, generated by the droplet method.

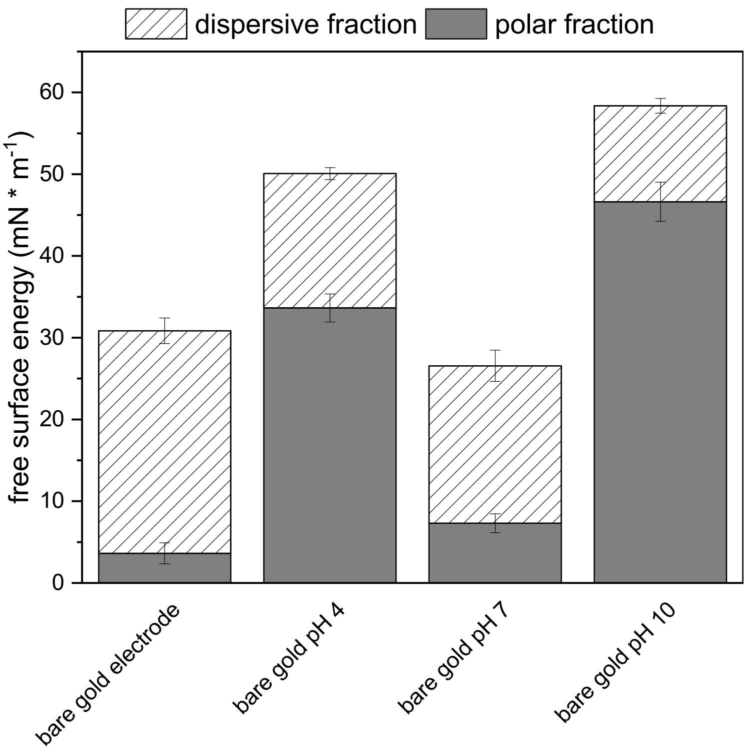

Figure S5. Surface free energy of the bare gold electrode and the bare gold electrode stored in different pH solutions.

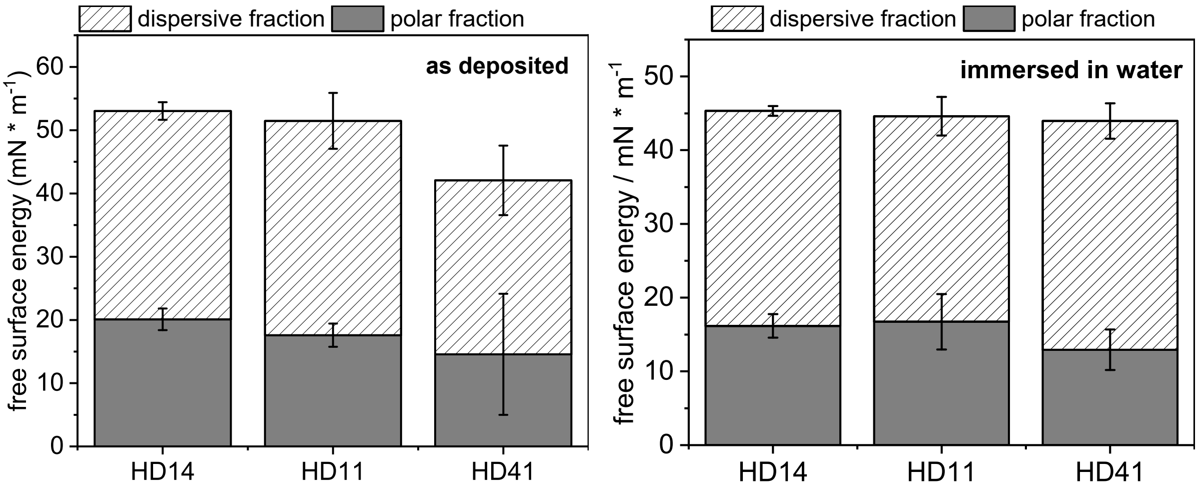

Figure S6. Surface free energy of the hydrogels mixtures generated by the nebulizer method (left: as-deposited, right: after immersion in water for 24 h) divided into dispersive and polar fraction.

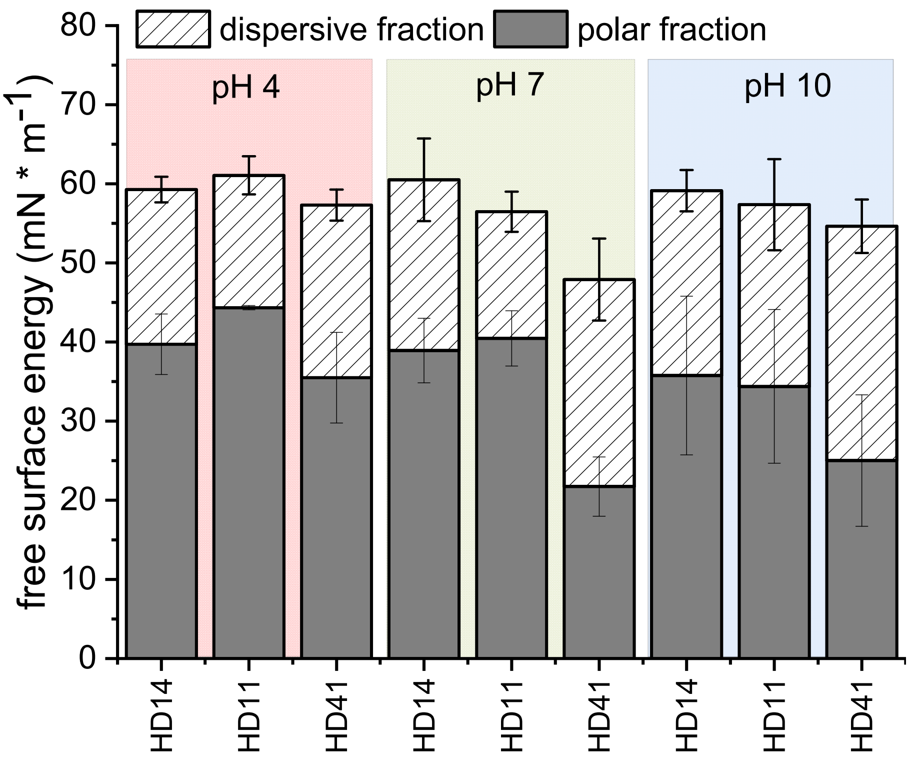

Figure S7. Surface free energy of the n-HD mixtures dried from different pH solutions.

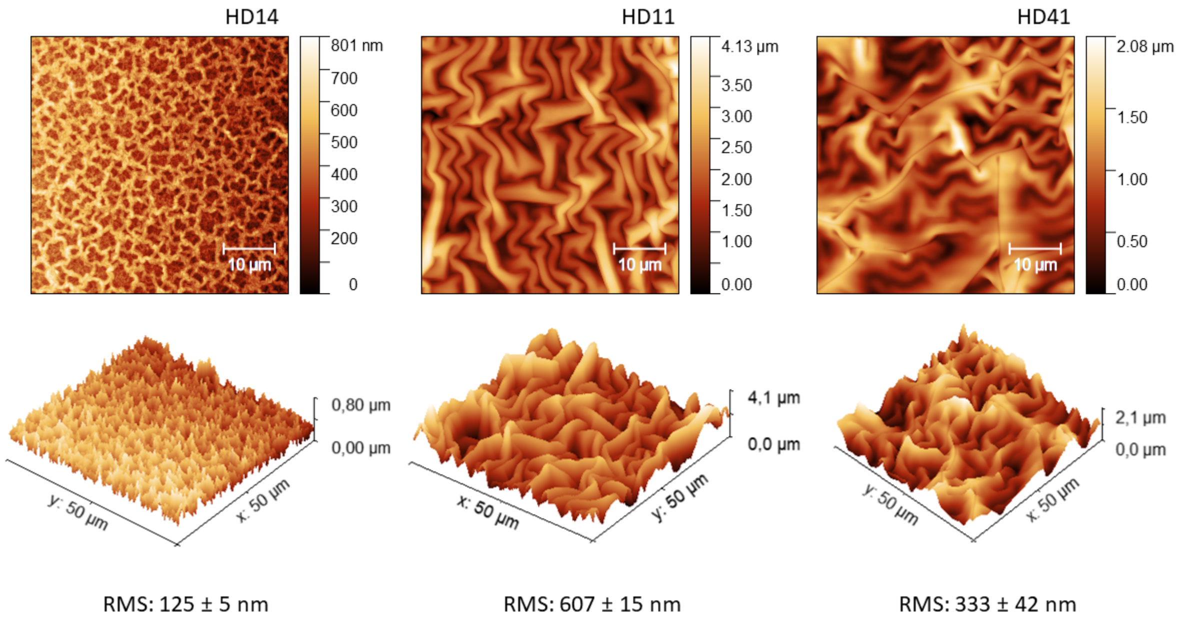

Figure S8. AFM images of the plasma-polymerized hydrogel coatings d-HD14, d-HD11, d-HD41 deposited on a smooth glass substrate.

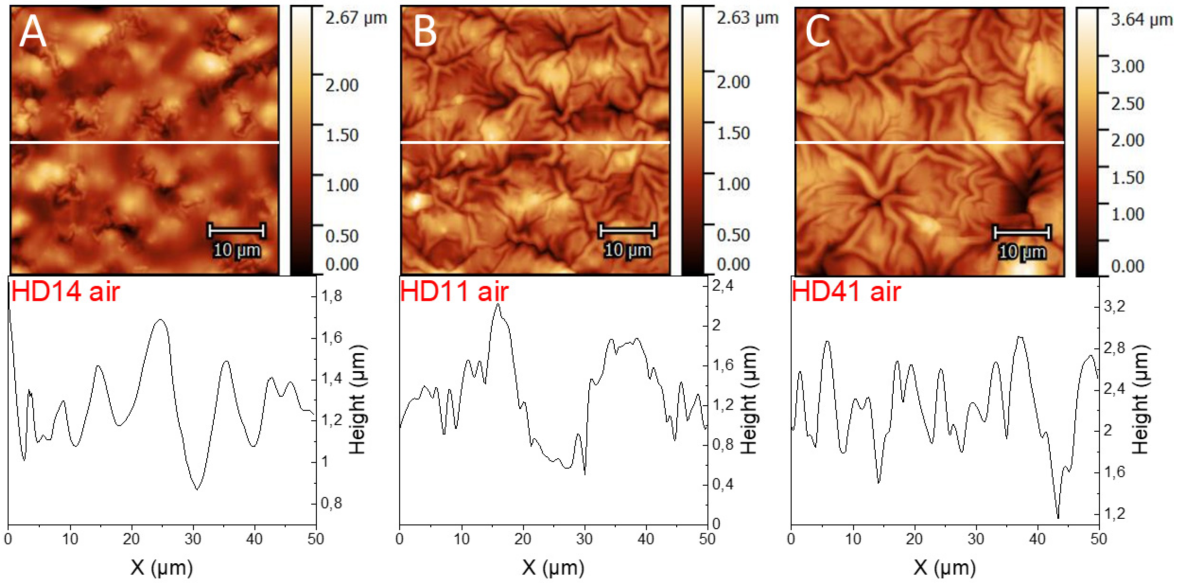

Figure S9. AFM images and the corresponding height profiles of the plasma-polymerized hydrogel coatings d-HD14 (A), d-HD11 (B) d-HD41 (C) deposited on gold electrodes.

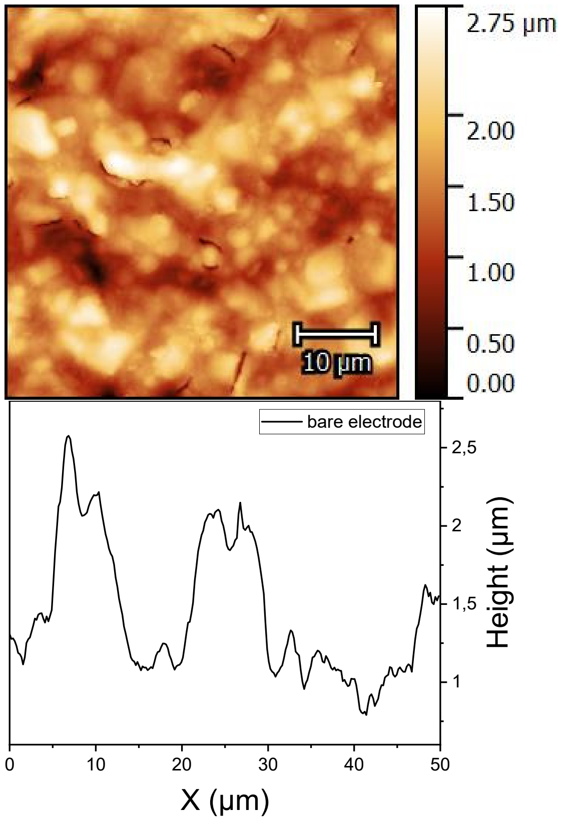

Figure S10. AFM image and the corresponding height profile of the bare gold electrode.

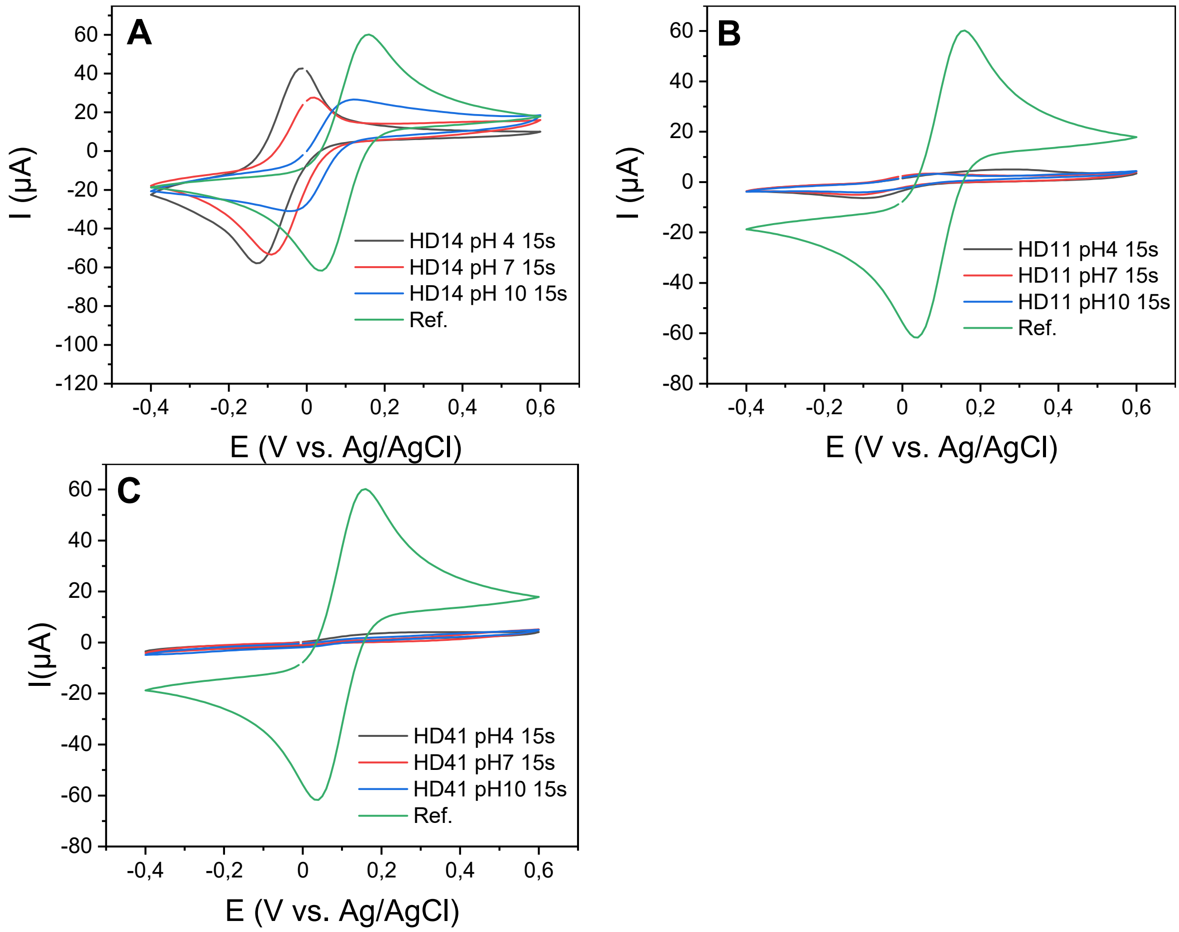

Figure S11. Cyclic voltammograms (A-C) of the plasma polymerized hydrogel mixtures (generated by the nebulizer method) in different pH buffer solutions.
